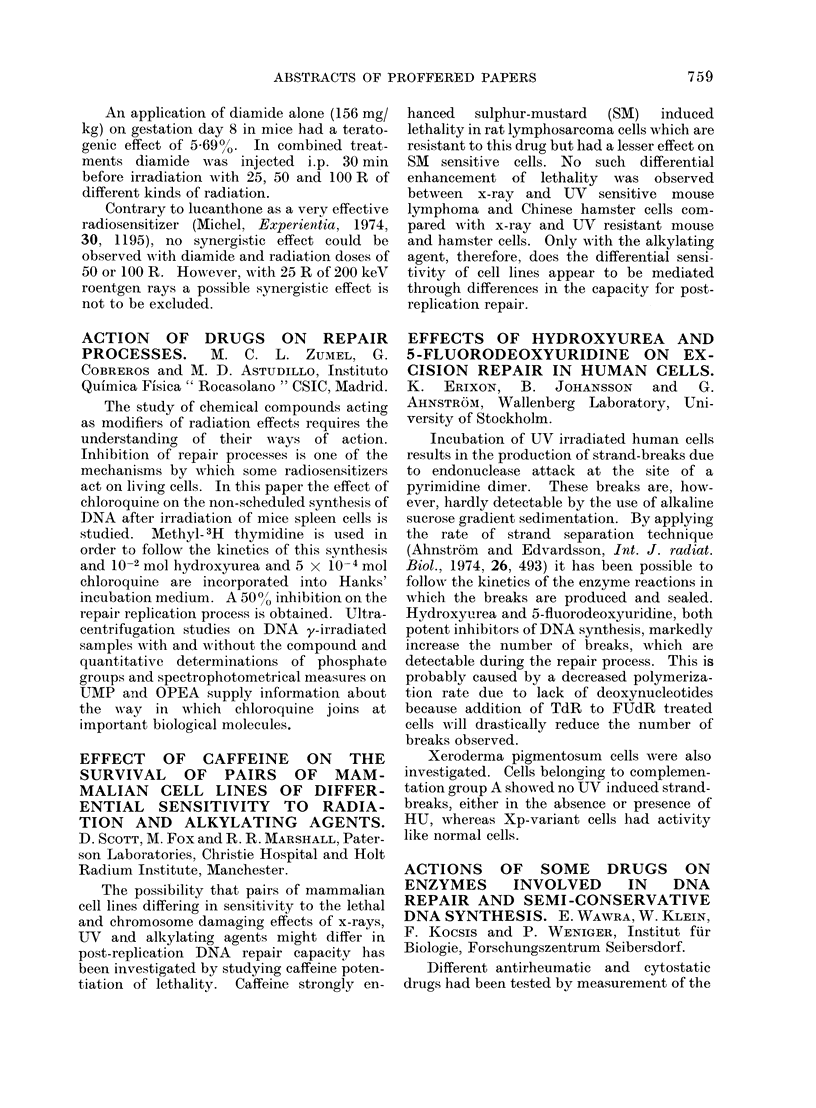# Proceedings: Effects of hydroxyurea and 5-fluorodeoxyuridine on excision repair in human cells.

**DOI:** 10.1038/bjc.1975.317

**Published:** 1975-12

**Authors:** K. Erixon, B. Johansson, G. Ahnström


**EFFECTS OF HYDROXYUREA AND 5-FLUORODEOXYURIDINE ON EXCISION REPAIR IN HUMAN CELLS.** K. Erixon, B. Johansson and G. Ahnström, Wallenberg Laboratory, University of Stockholm.

Incubation of UV irradiated human cells results in the production of strand-breaks due to endonuclease attack at the site of a pyrimidine dimer. These breaks are, however, hardly detectable by the use of alkaline sucrose gradient sedimentation. By applying the rate of strand separation technique (Ahnström and Edvardsson, *Int. J. radiat. Biol.*, 1974, **26**, 493) it has been possible to follow the kinetics of the enzyme reactions in which the breaks are produced and sealed. Hydroxyurea and 5-fluorodeoxyuridine, both potent inhibitors of DNA synthesis, markedly increase the number of breaks, which are detectable during the repair process. This is probably caused by a decreased polymerization rate due to lack of deoxynucleotides because addition of TdR to FUdR treated cells will drastically reduce the number of breaks observed.

Xeroderma pigmentosum cells were also investigated. Cells belonging to complementation group A showed no UV induced strand-breaks, either in the absence or presence of HU, whereas Xp-variant cells had activity like normal cells.